# Interface micromechanics of transverse sections from retrieved cemented hip reconstructions: an experimental and finite element comparison

**DOI:** 10.1007/s10856-012-4626-2

**Published:** 2012-06-08

**Authors:** Daan Waanders, Dennis Janssen, Sanaz Berahmani, Mark A. Miller, Kenneth A. Mann, Nico Verdonschot

**Affiliations:** 1 Orthopaedic Research Laboratory, Radboud University Nijmegen Medical Centre, P.O. Box 9101, 6500 HB Nijmegen, The Netherlands; 2 Department of Orthopaedic Surgery, SUNY Upstate Medical University, Syracuse, NY USA; 3 Laboratory for Biomechanical Engineering, University of Twente, Enschede, The Netherlands

## Abstract

In finite element analysis (FEA) models of cemented hip reconstructions, it is crucial to include the cement–bone interface mechanics. Recently, a micromechanical cohesive model was generated which reproduces the behavior of the cement–bone interface. The goal was to investigate whether this cohesive model was directly applicable on a macro level. From transverse sections of retrieved cemented hip reconstructions, two FEA-models were generated. The cement–bone interface was modeled with cohesive elements. A torque was applied and the cement–bone interface micromotions, global stiffness and stem translation were monitored. A sensitivity analysis was performed to investigate whether the cohesive model could be improved. All results were compared with experimental findings. That the original cohesive model resulted in a too compliant macromechanical response; the motions were too large and the global stiffness too small. When the cohesive model was modified, the match with the experimental response improved considerably.

## Introduction

Stable fixation at the cement–bone interface is essential for the longevity of cemented components used in cemented total hip arthroplasty, since aseptic loosening at the cement–bone interface is the main reason for revision surgery [1]. The polymethymethacrylate (PMMA) bone cement used in cemented hip reconstructions is usually not osteoconductive and therefore physicochemical bonding between the bone and cement cannot be expected [2, 3]. As a result, fixation between the bone and cement relies upon cement penetration into the bone [4] which results in a complex mechanical interlock between the two constituents [5]. However, this mechanical interlock can be considerably degraded after only 1 year in vivo service as a result of bone resorption [6–8]. This degradation weakens the cement–bone interface considerably relative to the direct post-operative situation [9] making the cement–bone interface one of the most compliant regions in cemented hip reconstructions [6].

In previous finite element analyses (FEA) of cemented hip reconstructions, the mechanical characteristics of the cement–bone interface have often been overly simplified. In several analyses the cement–bone interface was considered to act as (1) an infinitely stiff interface [10–12]; (2) a frictional contact layer [13, 14]; or (3) as a layer of soft tissue elements which represented osteolysis around the cement mantle [15, 16]. However, the validity of these three approaches to represent the interface mechanics is debatable. Experiments with laboratory prepared cement–bone interface specimens [17] showed a huge variation in stiffness and strength, which was not consistent with the three aforementioned assumptions.

A more appropriate approach to model the actual mechanical response of the cement–bone interface is through use of using cohesive zone models [18–21]. In these cohesive zone models a constitutive relationship has to be defined, which describes the interaction between the interface tractions and displacements in normal and shear direction [22]. Experiments in which cement–bone interface specimens are loaded in multiple directions could serve as an input for the cohesive zone models [23, 24]. However, the huge variation in mechanical responses due to interfacial variations makes it very difficult to develop a comprehensive cohesive zone model using an experimental approach. This is because each experimental specimen can only be loaded to failure in one direction, and the cohesive zone model requires a full description of the mixed-model failure response. An elegant alternative to study the mixed-mode failure response is the use of micromechanical FEA models [25]. Using this approach, a cohesive zone model has recently been developed in which the interfacial morphology was incorporated [26].

The cement–bone interface does not exhibit a homogenous morphology around the cement mantle [7], which subsequently results in local differences in mechanical characteristics. However, these local mechanical differences at the cement–bone interface have never been included in previous FEA studies. Moreover, previous macro FEA studies of cemented hip reconstructions which included cohesive zone models have never been directly validated with physical experiments. It has never been investigated whether a cohesive zone model of the cement–bone interface as determined on a micro level is directly applicable and yields appropriate results on a macro level.

The goal of this study was to investigate whether the micromechanical response of the cement–bone interface could be reproduced on a macro level by simulating macromechanical experiments [6]. A subsequent goal was to investigate how the micromechanical characteristics of the cement–bone interface influence the mechanical properties on a macro level. From two transverse sections of cemented hip reconstructions with considerable mechanical differences [6] FEA models were generated. The FEA models consisted of bone, the cement–bone interface, which was modeled by cohesive elements, a cement mantle and a stem. Like in the experiments, a torsional loading regime was applied to the stem while monitoring the motions at the cement–bone interface. Using this approach, we asked the following three research questions: (1) Can the motions that occurred experimentally at the cement–bone interface be reproduced? (2) Is the previously derived micromechanical mixed-mode formulation of the cement–bone interface directly applicable on a macro level? and (3) How do the micromechanics of the cement–bone interface influence the macromechanical properties of the complete reconstruction?

## Methods

### Specimen preparation

**Fig. 1:**
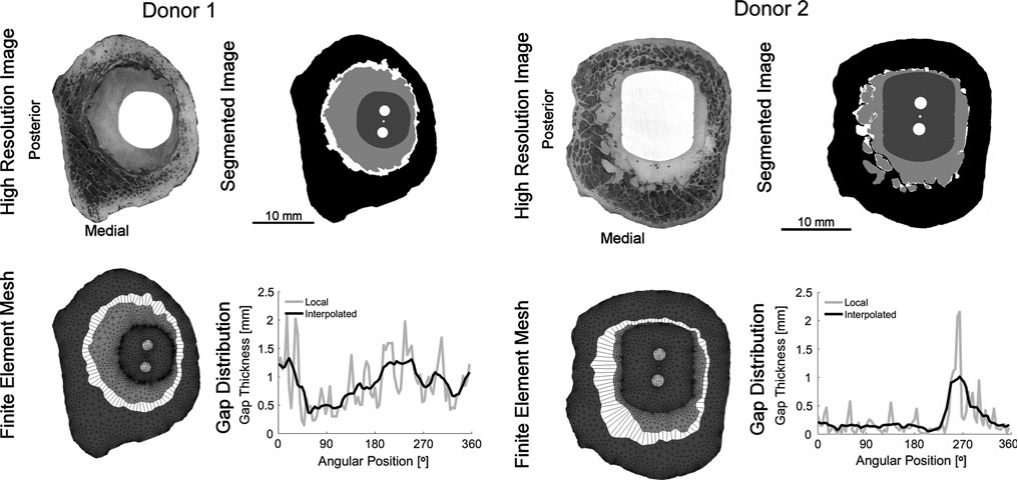
FEA modeling procedure of the two donors. High Resolution Image: in order to capture the morphology at the surface of each transverse section, a high-resolution image with pixel size of 5.7 μm was taken. The images were re-orientated such that the left side and bottom were the posterior and medial side, respectively. Segmentation: the high resolution images were segmented into six parts: (I) bone, (II) cement–bone gaps, (III) cement, (IV) stem-cement gaps, (V) stem and (VI) screw holes. The small dot between Finite Element Mesh: from each segmented transverse section an FEA mesh was generated. The bone, cement and stem were meshed with 2D plain strain triangles, while the complete cement–bone interface was meshed with 2D quad cohesive elements. All elements had an assumed thickness of 10 mm. Gap distribution: for each cohesive element of the cement–bone interface the average local gap thickness was calculated. Subsequently, the interpolated gap thickness was calculated by taking the average local gap thickness of the four adjacent elements on both sides of the considered element. Note that the mean gap thickness is the same for both cases

Two postmortem retrieved transverse sections of cemented hip reconstructions were considered for this study. The specimens were selected based on their mechanical response as determined by [6]: donor 1 and 2 (Table 1) were the most torsionally compliant and the stiffest specimen analyzed, respectively [6]. The considered transverse sections had a thickness of 10 mm and were retrieved from two different donors at autopsy (Table 1). The two donors were provided by the Anatomical Gift Program at SUNY Upstate Medical University [6]. Donations were made between 1 and 2 days after death and frozen at −20 °C prior to tissue harvest. Age, sex, number of years in service, cause of death, implant type and distance of the cut section from the calcar were documented. After mechanical testing of each transverse section, the surface roughness (Ra) of the stem was determined. By observing the porosity of the mid-mantle on the sectioned surface, it was assessed whether the cement was vacuum mixed. Planar x-rays of the cemented femur construct were made, after which it was assessed whether the cement–bone interface fixation loose or not loose (Table 1). A high-resolution image (pixel size: 5.7 μm) was made of each transverse section to document the morphology at the surface of the section (Fig. 1; High Resolution Image).

**Table 1:** Donor information for the two investigated cemented implants

	Donor 1	Donor 2
Age	85	67
Sex	Female	Female
Years in service	8	14
Cause of death	Bacterial endocarditis	Alzheimer’s disease
Implant type	Versys cemented—Zimmer	Harris precoat—Zimmer
Distance from calcar (mm)	40	30
Stem roughness (Ra, μm)	2.5	1.3
Vacuum-mixed	Yes	Yes
Radiographically loose	Yes	No
FEA model dimensions		
Number of elements	13,215	9,425
Number of nodes	7,271	5,234
Assumed friction coefficient at stem-cement interface	0.3	2.0

### Experimental testing

The protocol used for experimental testing of the transverse sections has been documented before [6] and will therefore only be described in brief. The outer surface of each transverse section was fixed in a custom-machined block. Subsequently, the stem of each transverse section was loaded by a torsional loading regime. The torque limits were set to 0.22 and 0.73 Nm in anteversion and retroversion, respectively, what represented torques that occur during normal walking [27]. During each loading cycle a digital image correlation (DIC) technique was used to quantify the motions at the cement–bone interface. The DIC sampling locations were placed at a distance of 0.25 mm from the interface to prevent errors in the DIC sampling at the material discontinuities. The angular rotation of the stem was also measured using DIC.

### FEA modeling

From each transverse slab a FEA model was generated. First, the high-resolution image was segmented into six parts: (I) bone, (II) cement–bone gaps, (III) cement, (IV) stem-cement gaps, (V) stem and (VI) screw holes (Fig. 1; Segmentation). The screw holes in the stem indentify the locations where the torque was applied. Next, the contours of the segmented bone, cement and stem were determined by a Moore Neighborhood algorithm. A Douglas–Peucker line simplification was subsequently applied to reduce the number of line segments of each contour [28]. Because of the physical thickness of the transverse sections (10 mm), the simplified contours were subsequently meshed with 2D plain strain triangles with an assumed thickness of 10 mm (Fig. 1; Finite Element Mesh). The cement–bone interface was meshed with 90 2D quad cohesive elements with a fixed 4 degrees of angular spacing. The cohesive elements captured the complete interdigitated zone of the cement–bone interface. The nodes of the cohesive elements matched the experimental DIC locations, which had an offset of 0.25 mm relative to the contact interface. The resulting models contained on average 11,700 elements and 6,500 nodes (Table 1). Contact between the stem and the cement was modeled using a double-sided node-to-surface contact algorithm (MSC.MARC 2007r1, MSC Software Corporation, Santa Ana, CA, USA). The assumed friction coefficient of the stem-cement interface of donor 1 was set to 0.3 [13] and the precoated interface of donor 2–2.0.

### Boundary conditions

**Fig. 2:**
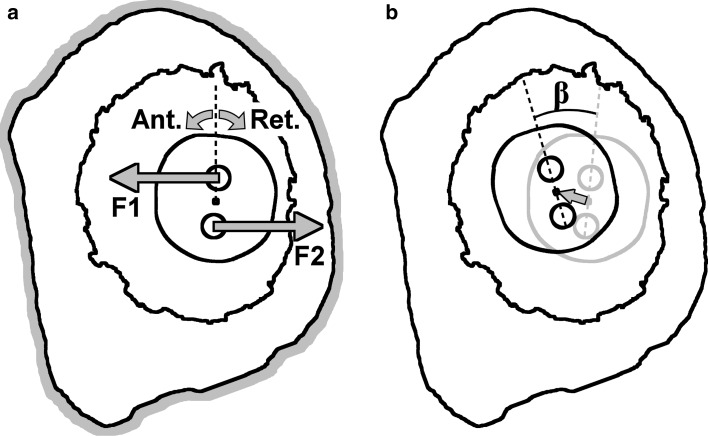
**a** The outside of the bone was fixed in all degrees of freedom. Two point loads, F1 and F2, were applied to the nodes in the middle of the two screw holes in order to rotate the stem in anteversion and retroversion. **b** The center of the stem was not fixed. The resulting displacement of the center of the stem was monitored as well as the angular rotation, β

To simulate the experimental setup, all the nodes on the outside of the bone were fixed in all degrees of freedom (Fig. 2a). Furthermore, an incremental point load was applied to the nodes in the centroid of the two screw holes to reproduce the torque (Fig. 2a). The resulting total torque was calculated for each increment. Like in the experiments, the FEA models were loaded up to 0.22 and 0.73 Nm in anteversion and retroversion, respectively. Although in the experiment the stem was only meant to rotate [6], small planar movements were measured during the loading cycles. Hence, in the current study the center of the stem was not fixed and had therefore the freedom to translate (Fig. 2b).

### Material properties

The stem, cement and bone were modeled as isotropic linear elastic materials. The stem was given an assumed Young’s modulus (E) and Poisson’s ratio (ν) of 210,000 MPa and 0.3, respectively [10]. Since the exact material properties of the cement were unknown, E and ν were taken as 3,000 MPa and 0.3, respectively [29–31]. In order to determine the material properties of the bone, the 2D FEA mesh of the bone was mapped back onto the high resolution image. Next, for each triangular element the average gray value was determined based on the 8-bit grayscale of the high resolution image (Fig. 1). The material properties of the bone were assumed to be linearly dependent on the average gray value [29]. The lowest and highest gray value elements were assigned a Young’s modulus of 0.1 and 20,000 MPa, respectively.

### Cohesive modeling cement–bone interface

The mechanics of the cement–bone interface were modeled using a recently developed cohesive model [26]. This cohesive model described the elastic behavior of the cement–bone interface in multiple directions. It determined the interfacial tractions [MPa] in normal and tangential direction (*T*_*N*_ and *T*_*T*_) based on the interfacial displacements [mm] in normal and tangential direction (Δ_*N*_ and Δ_*T*_) and the interface morphology. The interface morphology was expressed by the gap thickness, GT, which defined the average gap between the cement and the bone. The tractions in normal and tangential direction were defined as:

1 $$ \left[ {\begin{array}{*{20}c} {{\text{T}}_{\text{N}} } \\ {{\text{T}}_{\text{T}} } \\ \end{array} } \right] = 10^{{A \cdot {\text{GT}} + B \cdot \frac{{\Updelta_{\text{N}} }}{\Updelta } + C}} \cdot \left[ {\begin{array}{*{20}c} {\Updelta_{\text{N}} - {\text{D}} \cdot \frac{{\Updelta_{\text{T}}^{2} }}{\Updelta }} \\ {\Updelta_{\text{T}} \left( {1 + {\text{D}} \cdot \frac{{\Updelta_{\text{N}} }}{\Updelta }} \right)} \\ \end{array} } \right] $$

in which

2 $$ \Updelta = \sqrt {\Updelta_{\text{N}}^{2} + \Updelta_{\text{T}}^{2} } $$

In this set of equations the term

3 $$ 10^{{A \cdot {\text{GT}} + B \cdot \frac{{\Updelta_{\text{N}} }}{\Updelta } + C}} $$

was defined as the stiffness parallel to the loading direction. The parameter values ‘*A*’, ‘*B*’, ‘*C*’ and ‘*D*’ were estimated from a series of computational cement–bone interface models which were loaded to failure in multiple directions while monitoring the interfacial tractions [26]. In the original description of the cohesive model, the estimated parameters ‘*A*’ and ‘*C*’ were used to express the response in pure tension and were estimated to equal—6.369 and 2.439, respectively. Parameter ‘*B*’ was used to incorporate the effect of the loading angle and was estimated to equal −0.298. Finally, parameter ‘*D*’ was used to define tractions perpendicular to the loading direction and was estimated to equal 0.316.

### Local gaps and interpolated gaps

In order to use the cohesive model properly, the gap thickness of each cohesive element in the cement–bone interface had to be determined. Therefore, each cohesive element was mapped back onto the segmented image, after which the local gap thickness, LGT, was calculated. LGT was defined as the average interface gap of the cement–bone interface within each individual cohesive element (Fig. 1; Gap Distribution). However, the width of the cohesive elements as used in the current study was on average a factor 9 smaller (0.79 mm) relative to the average width of the models used to determine the cohesive model (7.54 mm) [26]. Therefore, in order to study a possible mesh dependency, the local gap thickness was interpolated what resulted in the interpolated gap thickness, IGT. IGT was based on the LGT of the four adjacent elements on both sides of the considered element (Fig. 1; Gap Distribution):

4 $$ {\text{IGT}}_{\text{N}} = \frac{1}{9}\sum\limits_{i = - 4}^{4} {{\text{LGT}}_{N + i} } . $$

This resulted in a gap thickness for each element based on an imaginary width similar to the models of Waanders et al. [26]. Note that the mean gap thickness over the whole cement–bone interface is the same for both the interpolated as the local gap description.

### Sensitivity analysis

**Fig. 3:**
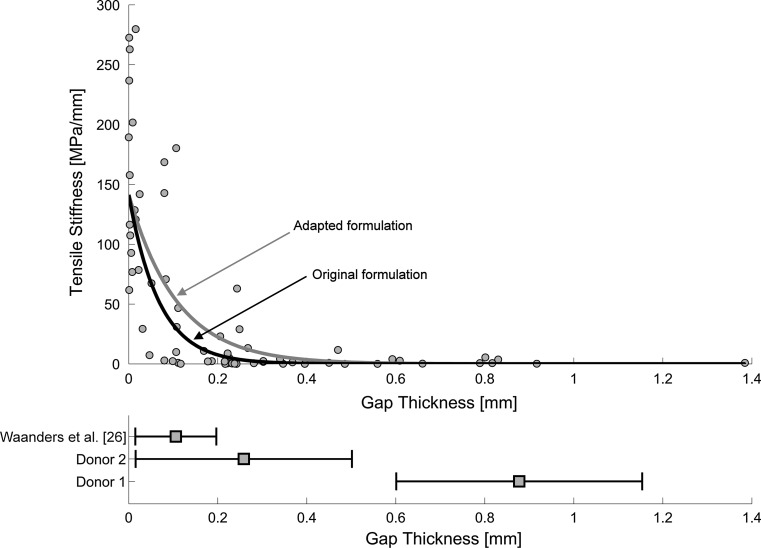
The *gray dots* in the upper graph presents the relationship between tensile stiffness and gap thickness as previously been found experimentally [9, 17]. The *solid black line* represents the tensile stiffness as a function of the gap thickness as determined by the developed cohesive model in pure tension (*A* = −6.369; *C* = 2.439; Δ_T_ = 0; Δ/Δ_N_ = 1) [26]. When a gap thickness of 0 mm was considered the cohesive model resulted in a tensile stiffness of 141.3 MPa/mm, which was much smaller than the average 229.5 MPa/mm (SD = 144.7) as found experimentally. The *gray line* represents the adapted formulation of the cement–bone interface (*A* = −4.000; *C* = 2.439). Note that the adapted formulation results in a higher tensile stiffness for larger gaps. The lower graph presents the variation in gap thickness over the two donors and the study of Waanders et al. [26]. Note that the gap thickness of donor 1 is very large relative to the considered range in gap thickness of Waanders et al. [26]

Limitation from the previously developed cohesive model was that it was based on four micromechanical FEA models with an average gap thickness of 0.106 mm (SD = 0.091 mm). When the gap thickness becomes considerably larger, like donor 1, the estimated stiffness might become too small relative to experimental findings [9, 17] (Fig. 3). Furthermore, the developed cohesive model resulted in a tensile stiffness of 141.3 MPa/mm when a gap thickness of 0 mm was considered. This was much lower than what has been found experimentally: 229.5 MPa/mm (SD = 144.7; Fig. 3). Therefore, in the current study an additional sensitivity analysis was performed in which the parameters ‘*A*’ and ‘*C*’ were varied. Parameter ‘*A*’ was considered to be −6.369, −5.0, −4.0, −3.0 and −2.0, while for parameter ‘*C*’ the values 2.439 and 2.650 were taken (Table 2), which corresponded to an initial tensile stiffness of 141.3 and 229.5 MPa/mm.

**Table 2:** Results of the sensitivity analysis in order to improve the mechanical response of the cement–bone interface

Test			θ [deg]		*r* [−]		$\overline{\Updelta }$ [mm]		D*r* [−]		${\text{D}}\overline{\Updelta }$ [−]		D [−]
			Donor 1	Donor 2	Donor 1	Donor 2	Donor 1	Donor 2	Donor 1	Donor 2	Donor 1	Donor 2	
	Experiment		65.8	235.5	0.04704	0.00085	0.6808	0.0024					
	A	C											
1*	−6.369	2.439	226.7	299.5	2.1668	0.0018	7.0848	0.0082	0.98	0.52	0.90	0.71	2.18
2	−5.000	2.439	238.2	296.3	0.6991	0.0013	2.5580	0.0055	0.93	0.35	0.73	0.56	1.73
3	−4.000	2.439	250.6	295.6	0.1987	0.0010	0.7301	0.0040	0.76	0.16	0.07	0.40	0.75
4	−3.000	2.439	252.1	293.6	0.0408	0.0008	0.1606	0.0029	0.15	0.12	3.24	0.17	2.10
5	−2.000	2.439	248.7	290.4	0.0063	0.0005	0.0296	0.0020	6.42	0.67	22.00	0.20	85.32
6	−6.369	2.650	230.5	300.5	2.5165	0.0014	8.3353	0.0053	0.98	0.38	0.92	0.55	1.96
7	−5.000	2.650	244.1	298.4	0.4604	0.0010	1.6834	0.0035	0.90	0.12	0.60	0.31	1.25
8	−4.000	2.650	251.0	297.3	0.1247	0.0008	0.4542	0.0026	0.62	0.09	0.50	0.08	0.80
9	−3.000	2.650	251.6	293.7	0.0253	0.0006	0.0990	0.0019	0.86	0.52	5.88	0.26	6.35
10	−2.000	2.650	246.3	289.4	0.0040	0.0004	0.0182	0.0013	10.88	1.18	36.41	0.85	223.18
		MEAN	244.0	295.5	0.6242	0.0009	2.1153	0.0037	2.35	0.41	7.12	0.41	32.56
		STD	9.2	3.7	0.9362	0.0004	3.0746	0.0021	3.49	0.34	12.25	0.25	71.92

*θ* was the mean angle with the concentration of largest micromotions. D*r* and ${\text{D}}\overline{\Updelta }$ were the relative difference between the FEA predicted and experimental value of *r* and $\overline{\Updelta }$, respectively (Eqs. 11 and 12), and D was the overall difference (Eq. 13). Regarding parameter C: the value 2.439 was determined by Waanders et al. [32] and the value 2.650 based on experimental findings (Fig. 3). The parameters A and C of Test 3 showed the smallest overall difference, D, relative to the experimental results and was therefore used for the adapted model (Fig. 3)

* For Test 1, Donor 1 was loaded to 0.40 Nm in retroversion instead of 0.73 Nm

### Output measures

Throughout the whole simulation the interface micromotions at the cement–bone interface were calculated. The micromotions consisted of a normal and shear component and the total interface micromotions were calculated as the vector sum of both components. Cumulative frequency distributions of the micromotions were generated for each donor specimen.

In order to study the effect of the utilization of interpolated gaps relative to local gaps, the total interface micromotions were analyzed for both cases. Additionally, the interfacial work of separation, W_sep_, at the cement–bone interface was determined. The work of separation was defined as the total amount of energy dissipated due to deformation of the interface [26]:

5 $$ {\text{W}}_{\text{sep}} = \int {{\text{T}}_{\text{N}} } \left( {\Updelta_{\text{N}} ,\Updelta_{\text{T}} } \right)\partial \Updelta_{\text{N}} + \int {{\text{T}}_{\text{T}} } \left( {\Updelta_{\text{N}} ,\Updelta_{\text{T}} } \right)\partial \Updelta_{\text{T}} $$

The first term in this work of separation expression was the work done by the normal traction, while the second term was the work done by the tangential traction.

As mentioned in the previous section, the center of the stem was not fixed in the FEA simulations what subsequently could result in a stem translation (Fig. 3b). Translations of the center of the stem in *x*- and *y*-direction were monitored and the total translation of the stem was calculated as the vector sum of both components. Finally, the global stiffness, *K*_glob_, [Nm/deg] of the whole FEA-model was calculated:

6 $$ {\text{K}}_{\text{glob}} = \left| {\frac{{{\text{M}}_{\text{ant}} - {\text{M}}_{\text{ret}} }}{{\beta_{\text{ant}} - \beta_{\text{ret}} }}} \right|, $$

where *M*_ant_ and *M*_ret_ are the torques at full anteversion and retroversion, respectively, and β the corresponding angular rotations of the stem [6].

### Quantification micromotions cement–bone interface

In order to quantify the spatial dispersion of micromotions at the cement–bone interface for each transverse section, circular statistics was used [32]. Using circular statistics, the mean angle of micromotions on the circumference of the cement–bone interface could be determined, as well as a measure for the concentration of the micromotions. A circular statistics approach was used because the nature of the angular position data results in a repeating pattern such that a 0° angular position is the same as a 360° angular position.

A so-called second-order analysis was performed in which the total micromotion, Δ, at each angular position was used as a weight factor for all the data points [33]. In this case, the mean angle with the concentration of largest micromotions, *θ*, and the measure of dispersion of the micromotions, *r*, were determinable as:

7 $$ \theta = { \tan }^{ - 1} \frac{\text{Y}}{\text{X}} $$

and

8 $$ r = \sqrt {{\text{X}}^{2} + {\text{Y}}^{2} } $$

where *X* and *Y* were weighted using the total micromotion at angle a_*i*_:

9 $$ {\text{X}} = \frac{1}{n}\sum\limits_{i = 1}^{n} {\Updelta_{i} \cdot { \cos }\left( {{\text{a}}_{i} } \right)} $$

and

10 $$ {\text{Y}} = \frac{1}{n}\sum\limits_{i = 1}^{n} {\Updelta_{i} \cdot { \sin }\left( {{\text{a}}_{i} } \right)} $$

Note that *r* is dependent on the micromotions and should therefore be interpreted relative to the magnitude of the micromotions. Furthermore, it should be noticed that θ and *r* do not give an indication about the average magnitude of the micromotion at the cement–bone interface. Therefore the mean micromotion of all 90 data points, $\overline{\Updelta }$, was determined additionally. In order to find the optimal cohesive description of the cement–bone interface based on the output of the sensitivity analysis, the relative difference between the FEA predicted and experimental value of *r* and $\overline{\Updelta }$ were determined as:

11 $$ {\text{D}}r = \frac{{\left| {r_{\text{fea}} - r_{ \exp } } \right|}}{{r_{\text{fea}} }} $$

and

12 $$ {\text{D}}\overline{\Updelta } = \frac{{\left| {\overline{\Updelta }_{\text{fea}} - \overline{\Updelta }_{ \exp } } \right|}}{{\overline{\Updelta }_{\text{fea}} }} $$

respectively.

Note that the magnitude of the differences does not indicate whether the response was under or overestimated. If the FEA response was overestimated, the values of D*r* and ${\text{D}}\overline{\Updelta }$ could never exceed 1.00. On the other hand, an underestimation of the FEA response could result in differences much larger than 1.0. Finally, a measure of the overall difference, D, was determined as:

13 $$ {\text{D}} = \frac{1}{2}\sum\limits_{i = 1}^{2} {{\text{D}}r_{i} + {\text{D}}\overline{\Updelta }_{i} } + {\text{D}}r_{i} {\text{D}}\overline{\Updelta }_{i} , $$

in which ‘*i*’ represents the donor.

## Results

### Original description cement–bone interface; interpolated gaps

**Fig. 4:**
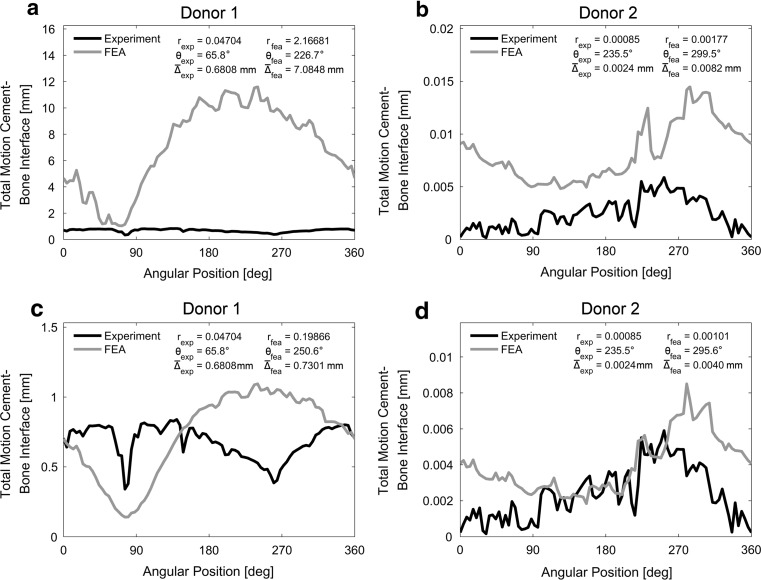
Distributions of the total motion along the circumference of the cement–bone interface when interpolated gaps were considered. **a** The response of donor 1 with the original description of the cement–bone interface (*A* = −6.369; *C* = 2.439) resulted in a too compliant interface. Both the dispersion of the micromotions, *r*, as the mean micromotion, $\overline{\Updelta }$, were overestimated. Note that for the experiment the value of *r* is relatively low and the significance of θ is debatable. **b** The response of donor 2 with the original description of the cement–bone interface (*A* = −6.369; *C* = 2.439) was also too compliant, but not as severe as donor 1. The distribution of micromotions showed the same trend, although there was a phase shift in *θ* relative to the experiment. **c** For donor 1, the adapted description of the cement–bone interface (*A* = −4.000; *C* = 2.439) showed a much better fit relative to the experiment. Note that the value of r for the FEA simulation is relatively large, which means that its dispersion of micromotions along the interface is not as arbitrary as in the experiment. **d** Also for donor 2 showed the adapted description of the cement–bone interface (*A* = −4.000; *C* = 2.439) a better fit with the experiment. Also here, there was no considerable change in *θ*

Using the original description of the mixed-mode mechanical response of the cement–bone interface (*A* = −6.369; *C* = 2.439; interpolated gaps), the responses of donor 1 and 2 were both too compliant relative to the experiments (Fig. 4a–b). Donor 1 could even not be loaded up to 0.73 Nm in retroversion and was therefore loaded with 0.4 Nm in this particular direction. Despite this torque reduction, donor 1 showed a considerable difference in the mean micromotion, $\overline{\Updelta }$, relative to the experiment which was overestimated by a factor 10 (${\text{D}}\overline{\Updelta }$ = 0.90; Table 2; Test 1). There was a considerable difference in angle with the concentration of largest micromotions, *θ*, between the experimental and FEA response for donor 1 (Fig. 4a). However, for the experiment the value of *r* was relatively low indicating that θ could not be properly determined. Although the distribution of the micromotions of donor 2 was qualitatively reasonable, there was a phase shift visible in the difference in θ between the experiment and FEA simulation (Fig. 4b).

### Original description; interpolated versus local gaps

**Fig. 5:**
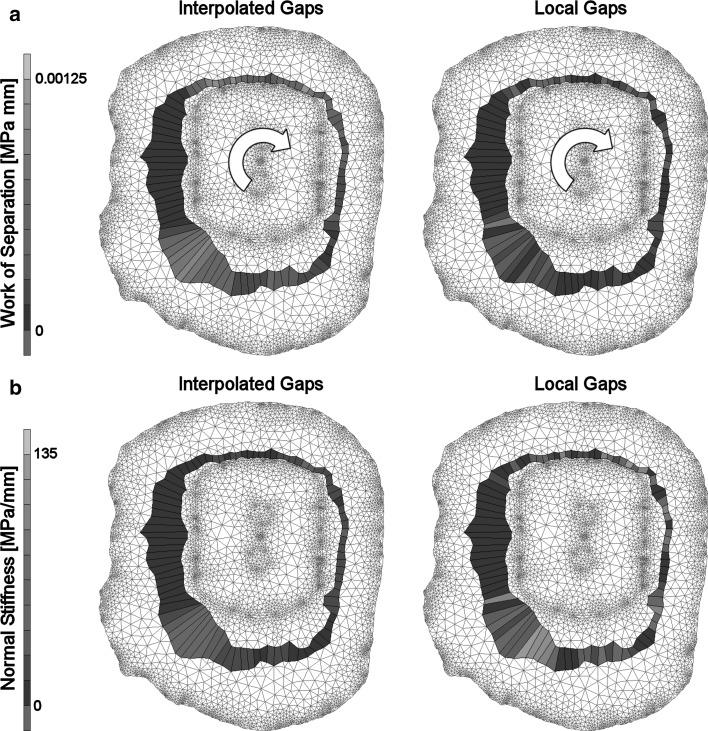
**a** When donor 2 was loaded to 0.73 Nm in retroversion, the simulation with interpolated gaps resulted in a smooth distribution of local work of separations at the cement–bone interface. When the stiffness of the cohesive elements was based on the local gaps, the distribution of local work of separations was irregular. Moreover, when local gaps were considered the total work of separation, W_sep_, was more than half the work of separation with interpolated gaps; 0.0264 versus 0.0585 MPa mm, respectively. **b** The distribution of the normal stiffness differed considerably between the two gap interpretations. This was a result of the stiffness formulation, which was exponentially dependent on the gap thickness

When the stiffness of the cement–bone interface (*A* = −6.369; *C* = 2.439) was based on local gaps, the magnitude of the simulated micromotions improved relative to interpolated gaps; the mean micromotion, $\overline{\Updelta }$, of donor 1 and 2 both decreased from 7.0848 and 0.0082 mm (interpolated gaps; Fig. 4a) to 1.1245 and 0.0038 mm (local gaps), respectively. A considerable difference in the work of separation, W_sep_, was determined between the two gap interpretations when the transverse section was loaded in full retroversion. For donor 1, W_sep_ was determined as 39.9378 and 2.4198 MPa mm for the interpolated and local gaps, respectively. For the interpolated and local gap interpretation of donor 2, W_sep_ was respectively determined as 0.0585 and 0.0264 MPa mm (Fig. 5a). Furthermore, the distribution of local work of separation was smooth when considering interpolated gaps and irregular when considering local gaps. This implies that when considering local gaps, the load transfer from the cement to the bone was concentrated on very specific locations.

### Sensitivity analysis

**Fig. 6:**
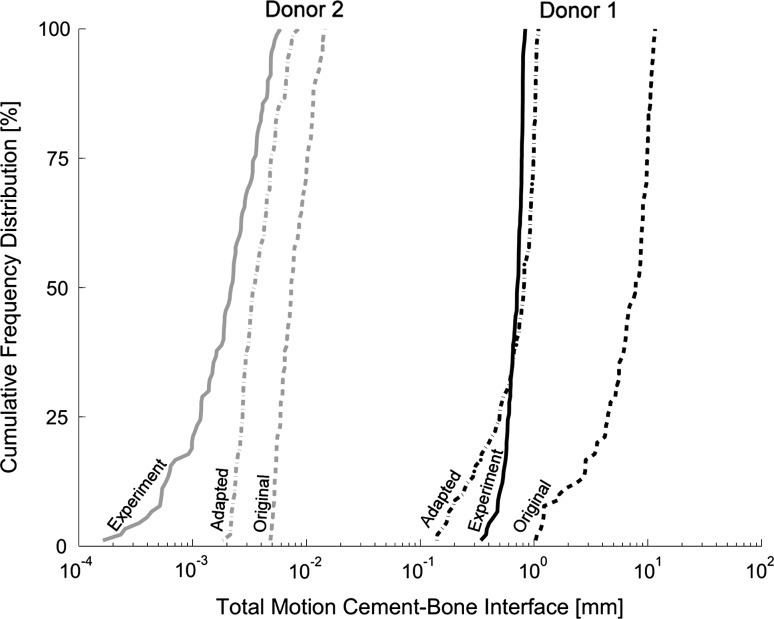
Cumulative Frequency Distribution of the total motion at the cement–bone interface are shown for donor 1 (*black*) and donor 2 (*gray*). The experimental distribution is indicated by a *solid line*, the original FEA (*A* = −6.369; *C* = 2.439) by a *dashed line* and the adapted FEA (*A* = −4.000; *C* = 2.439) by the *dash*-*dot line*

The sensitivity analysis showed that the angle with the concentration of largest micromotions, *θ*, hardly changed for both donor 1 (244.0° ± 9.2) and donor 2 (295.5° ± 3.7) (Table 2). Although Test 4 (*A* = −3.000; *C* = 2.439) showed the best responses for both donors in terms of D*r*, the corresponding ${\text{D}}\overline{\Updelta }$ for donor 1 was very large, subsequently making the overall difference, D, large too (Table 2). The parameters of Test 3 and 8 only differed in the value of C and they resulted in the smallest difference of all 10 tests. Since Test 3 was slightly better than Test 8, the parameters of Test 3 (*A* = −4.000; *C* = 2.439) were used for the adapted description of the cement–bone interface (Fig. 3). The main difference between the original and the adapted description of the cement–bone interface for donor 1 was the reduction of *r* and $\overline{\Updelta }$ (Fig. 4c) and for donor 2 the reduction of $\overline{\Updelta }$ (Fig. 4d). Independent on the angular position at the cement–bone interface, the distribution of total micromotions of the adapted description matched the experimental findings much better than the original distribution (Fig. 6).

### Stem translation

**Fig. 7:**
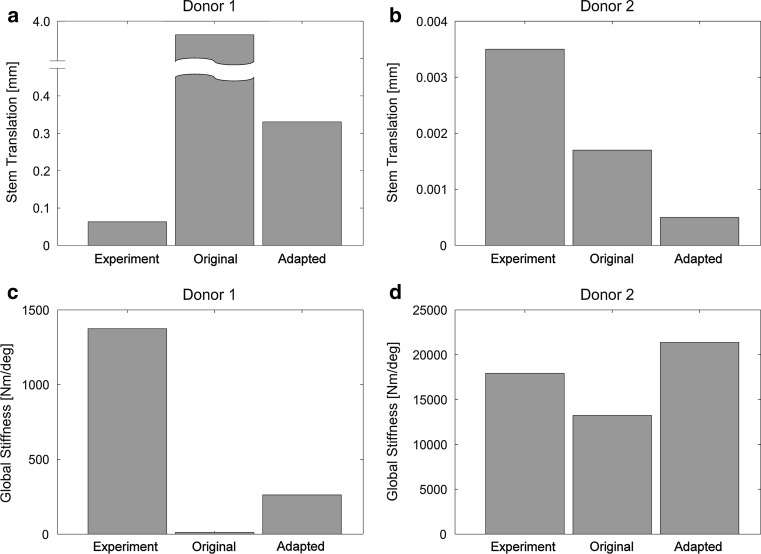
**a** The original description of the cement–bone interface (*A* = −6.369; *C* = 2.439) resulted in a excessive translation of the stem relative to the experiment. The translation of the stem of the adapted description (*A* = −4.000; *C* = 2.439) was much smaller, but still larger than in the experiment. **b** The translations of the stem were experimentally larger than in the FEA simulations. Also here, the adapted description of the cement–bone interface resulted in a smaller translation than the original description. However, the translation measured experimentally almost have the same magnitude as the RMS error of the DIC system (0.0026 mm) so the experimental translation could be misleading. **c** As a result of the large motions at the cement–bone interface of donor 1, the global stiffness with the original description of the cement–bone interface was extremely underestimated. The adapted description of the cement–bone interface did increase the global stiffness, but was still not in the range as determined experimentally. **d** The global stiffness of the original and the adapted description of donor 2 were under and over predicted, respectively

The original description of the cement–bone interface (*A* = −6.369; *C* = 2.439) resulted in an excessive stem translation in donor 1 (3.757 mm; Fig. 7a). The adapted description (*A* = −4.000; *C* = 2.439) reduced the stem translation considerably (0.331 mm), but was still larger than in the experiment (0.063 mm). For donor 2, the stem translation of the original cement–bone interface description (0.0017 mm) was three times the translation when considering the adapted description (0.0005 mm), but both much smaller than measured experimentally (0.0035 mm; Fig. 7b). However, the experimentally measured stem translations almost equal the RMS error of the DIC system (0.0026 mm) [6] and can therefore be misleading.

### Global stiffness

As a result of the large motions at the cement–bone interface of donor 1, the global stiffness with the original description of the cement–bone interface was extremely underestimated (12 Nm/deg) relative to the experiment (1,374 Nm/deg; Fig. 7c). After adaption of the interface, the global stiffness still did not reach the experimental global stiffness (265 Nm/deg). For donor 2 the predicted global stiffness fluctuated around the experimentally estimated stiffness (17,916 Nm/deg); 13,232 Nm/deg and 21,380 Nm/deg for the original and adapted description, respectively (Fig. 7d).

## Discussion

The main goal of the current study was to investigate whether the micromechanical response of the cement–bone interface could be reproduced on a macro level by the utilization of cohesive elements which were implemented in FEA models of transverse sections of postmortem retrieved cemented hip reconstructions. This study distinguishes itself from other FEA studies in which cohesive zone modeling was applied, because this is the first time the micromechanical based cohesive zone was directly compared to experiments on a macro level.

With respect to the first research question: when the cohesive zone formulation as determined by Waanders et al. [26] was considered, the determined mean micromotions, $\overline{\Updelta }$, at the cement–bone interface were too large for both donors (Table 3; Fig. 4). Donor 1 could not even be loaded up to the required 0.73 Nm in retroversion because of excessive interfacial deformations. Furthermore, not the exact distribution of micromotions was found along the circumference of the cement–bone interface, with donor 1 in particular (Fig. 4). However, when the cohesive zone formulation was adapted, the mean micromotions could be satisfactorily be reproduced, but the distribution of micromotions still remained an issue.

**Table 3:** Summery of the different descriptions

Description	*r* [−]		$\overline{\Updelta }$ [mm]		D [−]
	Donor 1	Donor 2	Donor 1	Donor 2	
Experiment	0.04704	0.00085	0.6808	0.0024	–
Original: interpolated gaps	2.16681*	0.00177	7.0848*	0.0082	2.18
Original: local gaps	0.33676*	0.00096	1.1245*	0.0038	1.06
Adapted	0.19866	0.00101	0.7301	0.0040	0.75

The original (*A* = −6.369; *C* = 2.439) with interpolated gaps resulted in a larger overall difference, D, than considering local gaps. The smallest overall difference, D, was obtained when considering the adapted description (*A* = −4.000; *C* = 2.439; interpolated gaps)

* Loaded to 0.40 Nm in retroversion instead of 0.73 Nm

With respect to the second research question in which we asked whether the cohesive formulation as determined by Waanders et al. [26] was directly applicable on a macro level we conclude that: (I) The determined cohesive formulation is too compliant, especially for gaps that are considerably larger than the gaps which were included in the original study [26], and (II) The way of gap implementation results in considerable mechanical differences.

Regarding (I) the underestimated stiffness for large gaps: the sensitivity analysis indicated that when the exponent which defined the reduction in stiffness as a result of an growing gap was decreased from −6.369 to −4.000, it matched the experiments considerably better (Table 3). Furthermore, we found that an increase of the stiffness considering a 0 mm gap thickness did not improve the response. This emphasizes that the imperfection of the original formulation lies in the range for large gaps. Additionally, the adapted cohesive model (*A* = −4.000; *C* = 2.439) has been re-analyzed in the regression model used by Waanders et al. [26]. It was found that the adapted cohesive model is still correlated to the mixed-mode responses as reported by Waanders et al. [26] (*r* ^2^ = 0.79; *P* < 0.001), hence it is still applicable for models with smaller gaps.

Regarding (II) the gap implementation: when the mechanics of the cement–bone interface were based on local gaps, rather than interpolated gaps, the motions at the cement–bone interface decreased considerably. This might be found remarkable since the mean gap thickness was the same for both cases. However, the interpolated gap description was a general smoothing of the coarse local gap distribution, leveling out all the local minimum and maximum gaps. The small local peak gaps had a substantial effect on the magnitude of the element stiffness, since it was exponentially dependent on the gap thickness (Fig. 5b). This can be seen in the work of separation, which was considerably smaller for the local gap description than for the interpolated description, although the local differences are much larger considering local gaps (Fig. 5a). Moreover, note that a refined cohesive mesh (e.g., 180 elements instead of 90) will stiffen the interface even more considering local gaps. As a result of the stiffening of the interface, the response with a local gap description matched the experimental response better than considering a interpolated gap description. However, we believe it is better to work with the interpolated description, provided that the adapted description of the cement–bone interface is used (Table 3). In the micromechanical mixed-mode study on which the cohesive zone formulation was based [26], local interface phenomena were neither taken into account. Only the apparent response of the complete structure was considered, making the formulation mesh size dependent.

With respect to the third research question, the mechanics of the cement–bone interface had a considerable effect on the macromechanical properties of the whole transverse section. The adapted description of the cement–bone interface decreased the stem translations considerably and increased the global stiffness, relative to the original description. The stem translation of donor 1 was overestimated for both the original as the adapted description. This can be explained by the center of the stem which was not fixed in the FEA simulations. The overestimation of these stem translations in donor 1 might also have contributed to the underestimation of the corresponding global stiffness; the limited freedom of the stem in the experimental environment might not only have affected its translation, but also its rotation. However, the differences found in global stiffness might also be a result of the motions at the stem-cement interface, which have not been assessed.

The cement–bone interface was modeled by 90 cohesive elements with 4 degrees of angular spacing which captured the complete interdigitated region of the cement–bone interface. This was done in order to match the DIC measurement locations of the experiment. This modeling approach resulted in cohesive elements which all had approximately the same width, but differed considerably in height. This does not affect the mechanical response of the interface since cohesive elements are, in contrast to ‘regular’ elements, displacement driven and not strain driven. The element height is therefore a redundant parameter in the cohesive element description. This also makes cohesive element suitable to be implemented as zero thickness elements [34, 35].

Since the applied cohesive model describes the elastic behavior of the cement–bone interface, no failure of the cement–bone interface was considered. However, it was supposed including interfacial failure would not be necessary, because the transverse torque limits that were applied were based on torques that occur during normal walking [6, 27]. Recent research has shown that no instant failure of the cement–bone interface occurs during walking [18] and, moreover, no failure was found in the physical experiments of Mann et al. [6].

Because of the small stem-cement motions that were found experimentally [6], the stem-cement interface was assumed to be not bonded, although other studies have assumed the opposite [36]. Since the friction coefficient at the stem-cement interface was unknown for both donors, they had to be assumed. Donor 1 was implanted with a Versys cemented stem which was assumed to have the same surface texture as a Charnley stem. Therefore, the friction coefficient was set to 0.3 for this donor [13]. Donor 2 was implanted with a Harris Precoat stem. During a post-experimental evaluation of the stem-cement interface no debonding was seen and, moreover, a considerable force was required to remove the stem from the cement mantle. However, since motions were found experimentally, the stem-cement interface was assumed to be not bonded and, therefore, was assigned a high friction coefficient of 2.0. To what extent the experimental reported motions could be assigned to material deformation is unknown. As mentioned before, misinterpretations at this interface could have affected the global stiffness of the transverse section.

There were several limitations that need to be acknowledged and addressed regarding this study. The first limitation lies within the modeling procedure that was used. Unfortunately, only surface information of the transverse sections was available. The transverse sections were too large for micro-CT scanning devices in order to document the complete internal 3D micro-morphology of the cement–bone interface. We therefore generated 2D models in which we assumed that the morphology of the cement–bone interface was homogenously distributed into the depth of the transverse section with the same gap distribution as visible on the outer surface. We are quite confident that fully 3D FEA models in which the gap distribution of the interior cement–bone interface is included would result in better responses. The ‘hidden’ internal morphology may weaken or stiffen the interface locally what may result in a better distribution of micromotion along the circumference of the cement–bone interface, since the current 2D models did not nicely match the experimental distribution. Another issue is the mesh size dependency of the utilized cohesive model. The way of implementing interface gaps into this model needs to be very well thought of in order to be consistent with the experimental input data [26].

Another limitation was that only two transverse sections were considered in this study, because the FEA modeling of the transverse sections was a highly time consuming process. However, the two analyzed transverse sections were selected based on their mechanical characteristics, which were the two most extreme as analyzed by Mann et al. [6]. We realize that more analyzed specimens would have strengthened the current study.

The fact that only the gap thickness was considered as a morphological parameter that influenced the stiffness of the cement–bone interface was another point of concern. Previous studies have shown that also other factors contribute to the mechanical response, such as a normalized cement–bone contact index or the contact area between the bone and the cement [5, 37].

From a clinical perspective, the results of the current study show there is a considerable difference in the macroscopic response of the cement–bone interface of well functioning cemented hip reconstructions. It is commonly known that degradation of the cement–bone interface can ultimately lead to aseptic loosening of the implant [38] and, moreover, it has recently been reported that an increased compliancy of the cement–bone interface also promotes cement-mantle failure [18]. A question that subsequently arises is whether the currently developed cohesive model is also applicable for clinical purposes. Could the cohesive model, for instance, be used for patient specific FEA models to investigate causes of early failure of the cemented reconstruction? Or is it applicable in the pre-clinical testing phase of newly developed orthopaedic implants? We realize the cohesive model should be further tested and optimized on a 3D level before it could be used for other purposes. Moreover, another restriction is that it is currently difficult to document the micro gap distribution at the cement–bone interface of a complete cemented hip reconstruction.

Based on the findings in the current study we conclude that with the current methods: (1) Only the mean micromotion and dispersion of micromotions as measured experimentally can be reproduced, but not the exact distribution of micromotions along the circumference of the cement–bone interface. (2) The previously derived micromechanical mixed-mode formulation is not directly applicable on a macro level. We also found that (3) the micromechanics of the cement–bone interface have a considerable influence on the macromechanical properties of the complete reconstruction. We finally conclude that, although the current study contributes to a better understanding in modeling the interfacial micromechanics of the cement–bone interface on a macro level, there are still lots to improve in terms of consistency of the cohesive formulation and modeling issues.
